# A Modified Intraperitoneal Chemotherapy Regimen for Ovarian Cancer: Technique and Treatment Outcomes

**DOI:** 10.3390/cancers13194886

**Published:** 2021-09-29

**Authors:** Ji Hyun Kim, Hyeong In Ha, Min Hae Kim, Mi Ra Han, Sang-Yoon Park, Myong Cheol Lim

**Affiliations:** 1 Center for Gynecologic Cancer, National Cancer Center, Goyang 10408, Korea; jihyunkim@ncc.re.kr (J.H.K.); mindview@ncc.re.kr (M.H.K.); parksang@ncc.re.kr (S.-Y.P.); 2 Department of Obstetrics and Gynecology, Pusan National University Yangsan Hospital, Pusan National University School of Medicine, Yangsan 50612, Korea; hi126908111@gmail.com; 3 Biostatistics Collaboration Team, National Cancer Center, Goyang 10408, Korea; hmr0209@ncc.re.kr; 4 Center for Clinical Trials, National Cancer Center, Goyang 10408, Korea; 5 Division of Tumor Immunology, Research Institute and Hospital, National Cancer Center, Goyang 10408, Korea; 6 Department of Cancer Control and Policy, Graduate School of Cancer Science and Policy, National Cancer Center, Goyang 10408, Korea

**Keywords:** intraperitoneal, chemotherapy, ovarian cancer, survival, toxicity

## Abstract

**Simple Summary:**

To overcome the limitations of intraperitoneal chemotherapy (IP), which include a low completion rate and port-related toxicities, we modified three institutional procedures concerning IP chemotherapy in patients with ovarian cancer: (i) insertion of an IP port in a neutral abdominal position, (ii) daily irrigation of the peritoneal cavity with warmed dextrose fluid (5%) for IP port patency and to prevent adhesion, and (iii) intravenous infusion of cisplatin on Day 2 after left colonic surgery. Among patients who underwent left colonic surgery, including low anterior resection, 27 were investigated to identify the rate of completion of six planned cycles and the feasibility of IP chemotherapy. With modifications in IP chemotherapy, the completion rate improved even after patients underwent left colonic surgery during cytoreduction with enhanced feasibility.

**Abstract:**

This study aimed to investigate treatment outcomes concerning three institutional modifications to intraperitoneal (IP) chemotherapy for patients with ovarian cancer. The medical records of 27 patients treated with IP chemotherapy were retrospectively reviewed. All patients had three IP chemotherapy institutional modifications; modified Gynecologic Oncology Group 172 regimen was used for the chemotherapy regimen. With institutional modifications, 63.0% (17/27) completed all six cycles of IP chemotherapy. Of the 17 and 10 patients with primary and recurrent ovarian cancer, respectively, 55.6% (15/27) underwent left colonic surgery, including low anterior resection. In patients with primary ovarian cancer, the IP chemotherapy completion rate was 76.5% (13/17). In patients with and without left colonic surgery, the IP chemotherapy completion rates were 53.3% (8/15) and 75.0% (9/12), respectively. No complications related to left colonic surgery during IP chemotherapy were identified. The most frequent grade 3–4 toxicities were gastrointestinal toxicities (33.3%) and neutropenia (29.6%). The median progression-free survival was 19.5 months in all patients and 25.2 months in patients with primary ovarian cancer. Three institutional modifications to IP chemotherapy increased the completion rate for planned IP chemotherapy, even after left colonic surgery. Further studies involving a larger study cohort are required to confirm survival outcomes using these modifications.

## 1. Introduction

Ovarian cancer is the leading cause of death in gynecologic cancer [1]. In the GLOBOCAN 2020 database, the number of new cases of ovarian cancer accounted for 1.6% of cancer cases worldwide and 2.1% of deaths. In women, ovarian cancer has been reported to be the eighth highest cause of death, followed by cancer of the cervix [2]. In Korea, the incidence of ovarian cancer has risen in recent decades, accounting for 2.9% of all cancers in women, and it is the second most common gynecologic cancer [3]. The standard treatment for ovarian cancer consists of optimal cytoreductive surgery and adjuvant chemotherapy. Although most patients undergo cytoreductive surgery followed by chemotherapy, the recurrence rate is also high.

Ovarian cancer mainly occurs within the peritoneal cavity, and then spreads to the lining surface of the peritoneum. Intraperitoneal (IP) chemotherapy has emerged as an effective treatment for locally advanced ovarian cancer [4]. Based on survival benefits identified in several randomized controlled trials, the National Comprehensive Cancer Network (NCCN) has recommended IP chemotherapy for patients with optimally debulked stage II–III disease [4,5,6]. However, in the Gynecologic Oncology Group (GOG) 172 study, the completion rate for planned IP chemotherapy was only 42% (86/205). Specifically, 24.3% (50/205) of patients underwent left colonic surgery, including low anterior resection, and the completion rate in patients with left colonic surgery was low (34%) [7]. Left colonic surgery was the only procedure in which several patients failed to initiate IP chemotherapy [7]. Additionally, Grade 3–4 gastrointestinal toxicity was high at 46% (94/205) with IP chemotherapy. Questions have been raised concerning chemotherapy toxicity, port-related complications, and their tolerability following the widespread use of IP chemotherapy [8].

To address toxicity issues, the Memorial Sloan Kettering Cancer Center (MSKCC) modified the GOG 172 regimen [9]. Using this modified GOG 172 regimen, the completion rate increased to 55% (56/102) and the incidence of gastrointestinal toxicity reduced by 8% (8/102) with comparable survival outcomes (median overall survival, 65.6 vs. 67.4 months) [9]. In that study, 40% (41/102) of patients who underwent colorectal resection were included; however, the number of patients with left colonic surgery and the completion rate specific to patients with left colonic surgery were not reported. Port-related complications and IP chemotherapy-related toxicities continue to impede the completion of the six planned cycles; hence, further improvement is required [7]. Using the well-established MSKCC regimen, we modified the application of IP chemotherapy in terms of port insertion, postoperative intraperitoneal hydration via an IP port to maintain port patency and to prevent intraperitoneal adhesion, and an alternative IV use of cisplatin on Day 2 after left colonic surgery at our institution. Therefore, this study aimed to investigate treatment outcomes in terms of the completion rate of the six planned cycles, complications related to IP chemotherapy, and survival after institutional modifications to IP chemotherapy.

## 2. Materials and Methods

Between January 2010 and May 2021, data concerning eligible patients with epithelial ovarian cancer were retrospectively identified and analyzed, as obtained from the clinical research data warehouse. Patients’ characteristics including age, FIGO stage at diagnosis, histologic type and grade, and residual tumor after cytoreductive surgery were examined.

**Figure 1:**
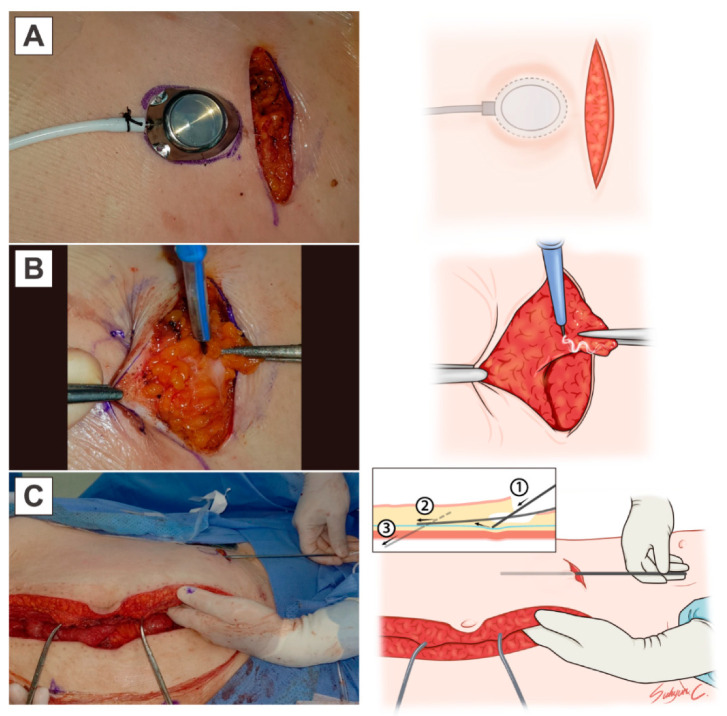
The modified IP catheter insertion technique: (**A**) A 5 cm length incision for the IP port was made at the mid-clavicular line on the lower rib, and the IP port chamber was outlined. (**B**) Subcutaneous tissue below the IP port chamber was partially removed for easy insertion of the needle into the IP port. (**C**) The catheter was inserted in a neutral position of the abdominal wall at the time of total closure of the wound. Retraction of the abdominal wall upward was avoided to prevent tortuous placement of the IP port line. While the guidewire was inserted into the fascia with the right hand, the left hand was used to palpate the optimal placement of the guidewire. The guidewire penetrated the fascia, muscle, and peritoneum subsequentially, while maintaining a natural position of the closed abdomen after passing 10–15 cm into the fascia.

Three institutional modifications were made to the application of IP chemotherapy. First, an IP port was inserted after cytoreductive surgery. An implantable intraperitoneal port (Celsite^®^, Inc. B. Braun, Germany) was placed below the lower margin of the right breast in the mid-clavicular line. The abdominal wall was in a neutral anatomical position, and traction of the abdominal fascia was obtained using a Kocher clamp, preventing acute-angulated insertion of the IP port into the abdominal wall (Figure 1). First, IP port patency was maintained by injecting 10 mL of heparin (125IU/1 mL) into the catheter to prevent postoperative IP port obstruction. Second, 200–400 mL of warmed 37 °C dextrose fluid (5%) was administered through the IP port daily, to fill the peritoneal cavity, preventing postoperative adhesion. Third, if the patient underwent left colonic surgery including low anterior resection, cisplatin was injected intravenously on Day 2 of the first chemotherapy cycle. The modified GOG 172 regimen was as follows: intravenous (IV) paclitaxel (135 mg/m^2^) over 3 h on Day 1, IP cisplatin (75 mg/m^2^) on Day 2, and IP paclitaxel (60 mg/m^2^) on Day 8, administered every 21 days for 6 cycles [9].

The primary endpoint of this study was the completion rate of the planned IP chemotherapy cycles. The number of completed cycles was recorded, and the reasons provided for discontinuing IP chemotherapy were reviewed. For the secondary endpoints, all recorded adverse events were graded using Common Terminology Criteria for Adverse Events (CTCAE) version 5.0, and complications regarding the IP port were also assessed, including catheter obstruction or malfunction and port site infection [4,7].

Progression-free survival (PFS) was defined as the time from IP port insertion after cytoreductive surgery until recurrence, death, or the date of the last contact. Overall survival (OS) was defined as the time from IP port insertion after cytoreductive surgery until death. Estimations of PFS and OS were determined after analyzing Kaplan–Meier curves.

## 3. Results

### 3.1. Patient Characteristics

In total, 27 patients who underwent IP chemotherapy involving three institutional modifications were included in this study. Patients’ descriptive characteristics are summarized in Table 1. The median patient age was 59 years. Twenty-four patients were classified as stage III according to FIGO 2014 staging, and three patients were classified at stage IV because of cardiophrenic lymph node metastasis. Twenty-five (92.6%) patients had high-grade serous carcinoma, and the ovary was the primary disease site for all patients involved. Seventeen (63.0%) patients had primary ovarian cancer and ten patients had recurrent ovarian cancer. Left colonic surgery, including low anterior resection, was performed in 15 (55.6%) patients.

### 3.2. Safety Outcomes and Completion Rates

The percentage of patients who adhered to the recommended IP chemotherapy modifications was 85.2%, as shown in Table 2. The modified GOG 172 regimen was used for all patients, and 96.3% of patients received warmed dextrose fluid (5%) into the peritoneal cavity via the IP port to prevent postoperative adhesion and to maintain IP catheter patency.

The completion rate of the six planned cycles of IP chemotherapy was 63.0% in the 27 patients. Of 27 patients who received IP chemotherapy, 8 (29.6%) patients completed ≤2 chemotherapy cycles. The IP chemotherapy completion rate did not differ statistically according to left colonic surgery (53.3% vs. 75.0%, *p* = 0.8691; Table 3). In 17 patients with primary ovarian cancer, 13 (76.5%) patients completed 6 cycles of IP chemotherapy compared with 42% in the GOG 172 regimen. Reasons for discontinuation of IP chemotherapy were as follows: severe gastrointestinal disorders including enterocolitis, ascites (n = 3, 11.1%), loss to follow-up during chemotherapy (n = 3, 11.1%), IP port obstruction (n = 2, 7.4%), IP port site infection (n = 1, 3.7%), and disease progression (n = 1, 3.7%). Among patients with primary ovarian cancer, a port-related complication was observed in one (5.9%) patient. 

In Table 4, severe or life-threatening adverse events (grades 3–4) were identified during IV/IP chemotherapy. Gastrointestinal disorders including colitis, constipation, and gastrointestinal perforation were the most frequent adverse events (33.3%). Neutropenia was observed in eight (29.6%) patients compared with 76% of patients using the GOG 172 regimen. Port-related complications including obstruction, infection, and leakage were identified in four (14.8%) patients compared with 26% in those using the GOG 172 regimen. No mortality was identified with IP chemotherapy.

### 3.3. Survival Outcomes

**Figure 2:**
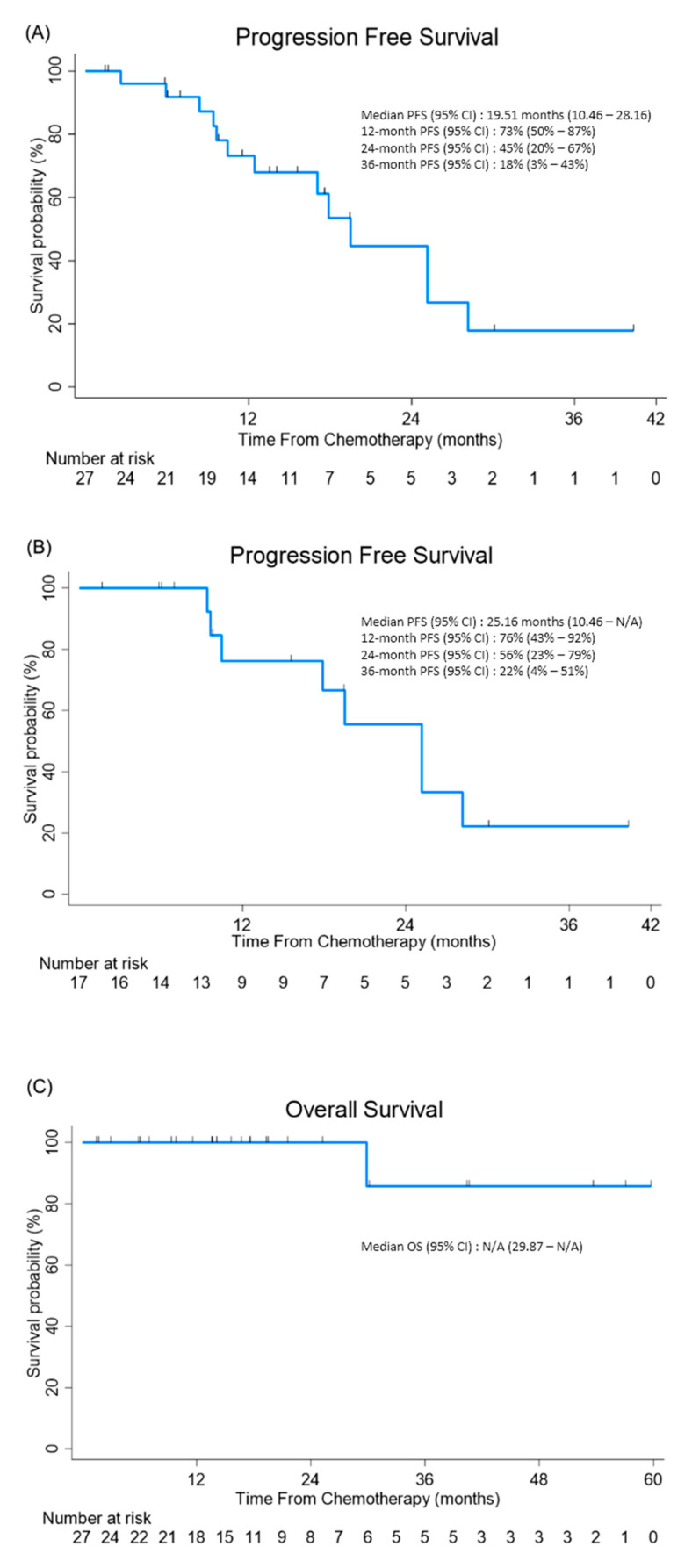
A Kaplan–Meier curve for progression-free survival and overall survival: (**A**) A Kaplan–Meier curve of progression-free survival in all patients’ post-IP chemotherapy. (**B**) A Kaplan–Meier curve of progression-free survival in patients with primary ovarian cancer post-IP chemotherapy. (**C**) A Kaplan–Meier curve of overall survival in all patients’ post-IP chemotherapy.

The median duration of follow-up was 20.7 months, with three patients lost to follow-up. The median PFS was 19.5 months (95% CI 10.46–28.16; Figure 2A). In patients with primary ovarian cancer, the median PFS was 25.2 months (95% CI 10.46–not assessable; Figure 2B). The median OS was not determined for one patient, who died due to cancer progression (95% CI 29.87–not assessable; Figure 2C). 

## 4. Discussion

Based on previous research and on our clinical practice experience of IP chemotherapy, prevention of IP port obstruction and improved tolerability of IP chemotherapy even after low anterior resection—which is a required surgical procedure for almost two-thirds of patients with advanced ovarian cancer—are essential for successful IP chemotherapy [10,11]. Complete cytoreduction using left colonic surgery and IP chemotherapy is important for patients with ovarian cancer; however, IP chemotherapy has not been found to be well tolerated in patients undergoing left colonic surgery, including low anterior resection [8,12,13]. In this study, the completion rates of patients in IP chemotherapy with institutional modification, who underwent left colonic surgery, were improved compared with previous publications. This finding needs to be evaluated in a larger prospective cohort study.

In terms of patient selection, we also included patients at stage IV, according to 2014 FIGO criteria, in this study. All three large randomized controlled trials in relation to IP chemotherapy (i.e., GOG 104, GOG 114, and GOG 172) used 1988 FIGO stages [4,5,6,12]. Therefore, stage IV splenic parenchymal metastatic ovarian cancer, according to 2014 FIGO stages, was reclassified as stage III according to 1988 FIGO stages, and was included in previous randomized trials of IP chemotherapy. Furthermore, cardiophrenic lymph node metastasis was surgically resected and pathologically evaluated after 2009 [13]. In daily clinical practice, the cardiophrenic lymph node was not critically considered before 2009. A significant proportion of patients with cardiophrenic lymph node metastasis findings, based on computed tomography findings, were classified as FIGO stage III disease during this period. Therefore, the three GOG randomized trials could have included patients with cardiophrenic lymph node metastasis as stage III, which had not otherwise been fully evaluated and considered at the time. Such patients could be treated with IP chemotherapy, especially in cases of complete surgical resection of parenchymal splenic metastasis or cardiophrenic lymph node metastasis.

In our study, IP chemotherapy was also applied to patients with platinum-sensitive ovarian cancer. Previous studies of IP chemotherapy in recurrent ovarian cancer showed an improved survival outcome compared with intravenous chemotherapy with acceptable morbidities [10,12]. Details of these IP chemotherapy studies in recurrent ovarian cancer are shown in Supplementary Table S1.

**Figure 3:**
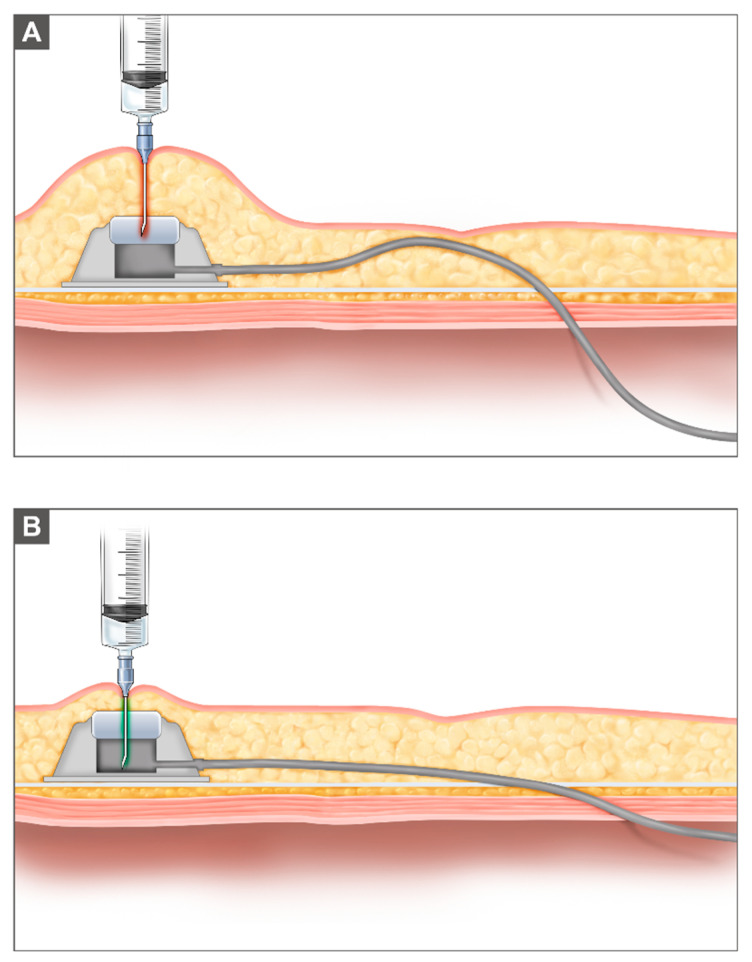
Placement of the IP port: (**A**) Prior to modification of the IP port insertion, the subcutaneous tissue on the IP port was not surgically reduced, and the IP port was inserted while upward retraction of abdominal wall was obtained using a Richardson retractor. Needling of the IP port was challenging because the subcutaneous tissue and tortuous insertion of the IP port line increased the risk of IP port obstruction. (**B**) After modification of the IP port insertion, the subcutaneous tissue on the IP port was surgically reduced for easy palpation and efficient needling of the IP port. The IP port line was straight onto the fascia and penetrated smoothly into the peritoneal cavity.

Despite technical improvements in the IP port, port-related complications remain a significant challenge in IP chemotherapy [14]. To ensure the successful completion of the six planned cycles of IP chemotherapy, proper insertion of the IP port is essential. When a catheter is tunneled subcutaneously into the peritoneal cavity, upward traction of the abdominal wall could be one factor in IP port obstruction (Figure 3). If the catheter is fenestrated into the peritoneal cavity running almost parallel to the neutral anatomical position of the abdominal wall, as illustrated in Figure 1, the likelihood of the catheter bending, or of blockage due to bowel movement, could be reduced (Figure 3). In addition, before the port is fixed on the inferior thorax at the mid-clavicular line, subcutaneous tissues over the IP port could be surgically removed for easy needling of the IP port. 

Finally, we consider that insertion of the IP port at the end of cytoreductive surgery is ideal for IP chemotherapy, enabling postoperative irrigation via the IP port and avoiding an additional operation for IP port placement. We did not fully evaluate postoperative adhesion prevention or the rate of IP port patency increase with postoperative irrigation via the IP port in this study; however, daily hydration of 200 mL 5% dextrose might potentially improve recovery and prevent postoperative adhesion. Recovery of bowel function by intraperitoneal irrigation and hydration via the IP port needs to be investigated in future studies.

Since colorectal surgery is frequently required in the surgical management of advanced stage ovarian cancer, careful management to prevent anastomotic leakage should be undertaken because adjuvant therapy is more likely to be delayed or cancelled due to complications related to anastomotic leakage [11,15]. There are limited data concerning the effect of IP chemotherapy on colonic anastomosis [16,17]. Experimental data with several cytotoxic drugs administered intraperitoneally showed adverse effects on anastomotic healing (Supplementary Table S2); however, intraperitoneal administration of paclitaxel was not found to be associated with colonic anastomotic leakage [16,18], and cisplatin had a negative effect on anastomotic wound healing [19]. Delays in anastomotic healing have different anti-proliferative effects, depending on each chemotherapy agent. Cisplatin has been found to have an inhibitory effect on fibroblast and endothelial cell proliferation, whereas no impairment in mucosal layer regeneration has been observed with paclitaxel [20]; therefore, delaying the IP injection of cisplatin might aid in the healing of colonic anastomosis.

This understanding is also supported by previous studies, which found that inserting a port at the same time as bowel surgery, and initiating IP chemotherapy after the first cycle was finished intravenously, did not increase the risk of major bowel complications [20,21]. With an alteration in the cisplatin administration route at the first cycle, the completion rate after left colonic surgery increased to 53.3% in this study compared with 34.0% using the GOG 172 regimen. Additionally, according to our institutional data for eight earlier cases without modifications, while three (37.5%) patients underwent left colonic surgery, they all failed to complete IP chemotherapy with cisplatin injected via the IP route in the first cycle. Concerning toxicities, 75% of patients complained of grade 3–4 abdominal pain or colitis when cisplatin was injected via the IP route. After cisplatin had been administered intravenously after left colonic surgery, grade 3–4 gastrointestinal-related complications were reduced to 33% in this study.

This study had several limitations. This was a retrospective study; therefore, some data were not consistent and some data were missing. Moreover, the data extracted from the earlier study period tended to be more limited than the data collected in the later period. Furthermore, this study comprised a relatively small sample size; thus, the statistical power to determine clinical outcomes was limited.

In conclusion, modifications to procedural techniques, in addition to a modified IP chemotherapy regimen, showed a better completion rate with propitious survival outcomes.

## Figures and Tables

**Table 1:** Patient and baseline disease characteristics.

Characteristics	Number of Patients (%)
	N = 27
Age (years)	
Mean ± SD (years)	60.9 ± 12.0
Median(min–max) (years)	59.0 (34.0–80.0)
FIGO surgical stage (FIGO stage 2014)	
IIIA	2 (7.4%)
IIIB	7 (25.9%)
IIIC	15 (55.6%)
IVB	3 (11.1%)
Histology (No.)	
High grade serous	25 (92.6%)
Endometrioid	0 (0.0%)
Clear cell carcinoma	0 (0.0%)
Mucinous	0 (0.0%)
Low grade serous	0 (0.0%)
Others	2 (7.4%)
Histologic grade	
Grade 1	1 (3.7%)
Grade 2	6 (22.2%)
Grade 3	20 (74.1%)
Primary disease site (No.)	
Ovary	27 (100%)
Peritoneum	0 (0.0%)
Fallopian tube	0 (0.0%)
Previous surgery	
Primary	
Primary cytoreductive surgery	13 (48.2%)
Interval debulking surgery	4 (14.8%)
Secondary	10 (37.0%)
Residual tumor	
R0 (no residual tumor)	23 (85.2%)
R1 (microscopic)	4 (14.8%)
R2 (macroscopic)	0 (0.0%)
Left colonic surgery	
No	12 (44.4%)
Yes	15 (55.6%)
FIGO, International Federation of Gynecology and Obstetrics	

**Table 2:** Adherence of institutional modifications and cycle completion.

Variables	N = 27
Institutional Modification	
Modified GOG 172 regimen	27 (100%)
Warmed dextrose IP infusion	26 (96.3%)
IV cisplatin Day 2 when left colonic surgery	23 (85.2%)
Cycle Completion–All	
Cycle ≤ 2 (number, %)	8 (29.6%)
2 < cycle < 6 (number, %)	2 (7.4%)
Cycle 6 (number, %)	17 (63.0%)
Cycle Completion–Primary	
Cycle ≤ 2 (number, %)	3 (17.7%)
2 < cycle < 6 (number, %)	1 (5.9%)
Cycle 6 (number, %)	13 (76.5%)
Reason for Discontinuation of IP chemotherapy–All	
Grade 3–4 Gastrointestinal disorder	3 (11.1%)
Loss to follow-up	3 (11.1%)
IP port obstruction	2 (7.4%)
IP port infection	1 (3.7%)
Disease progression	1 (3.7%)

**Table 3:** Cycles of IP chemotherapy when patient underwent left colonic surgery.

	Left Colonic Surgery		*p*-Value ^(1)^
No. of Cycles	No. (N = 12)	Yes (N = 15)	
1	2 (16.7%)	2 (13.3%)	0.8691
2	1 (8.3%)	3 (20.0%)	
3	0 (0.0%)	1 (6.7%)	
4	0 (0.0%)	0 (0.0%)	
5	0 (0.0%)	1 (6.7%)	
6	9 (75.0%)	8 (53.3%)	

^(1)^ Fisher’s exact test.

**Table 4:** Rate of grade 3–4 adverse events during treatment and port-related complications.

Adverse Event	N = 27
Anemia	0 (0.0%)
Neutropenia	8 (29.6%)
Thrombocytopenia	0 (0.0%)
aFever	6 (22.2%)
Gastrointestinal	9 (33.3%)
Infection	8 (29.6%)
Renal	1 (3.7%)
Metabolic	0 (0.0%)
Neurologic	0 (0.0%)
IP port related	
IP port obstruction	2 (7.4%)
IP port infection	1 (3.7%)
IP port leakage	1 (3.7%)
CTCAE 5.0	

## Data Availability

The data presented in this study are available on request from the corresponding author.

## Supplementary Materials

The following are available online at https://www.mdpi.com/article/10.3390/cancers13194886/s1, Table S1: Review of IP chemotherapy in patients with recurrent ovarian cancer, Table S2: Experimental data with several cytotoxic drugs administered intraperitoneally to identify the effect on anastomotic healing.
