# Supplementary material for: A Modified Intraperitoneal Chemotherapy Regimen for Ovarian Cancer: Technique and Treatment Outcomes
Source: Cancers (Basel). 2021 Sep 29;13(19):4886. doi: 10.3390/cancers13194886 (PMC8508040; doi:10.3390/cancers13194886)
Supplement: Supplementary file 1 [file cancers-13-04886-s001.zip › cancers-1372277-supplementary.pdf]

cancers

Table S1. Review of intraperitoneal chemotherapy in recurrent ovarian cancer.

No.	Authors, Year	Enrolled patients	Platinum sensitivity	Pathology	Study Design	Drugs	Completion rate	Survival	Port related Complications
1	Skaznik-Wikiel et al, 2012	56	Platinum sensitive	Serous (84.9%)	Retrospecitve, Single arm	1) IV paclitaxel 135mg/m2 for 3h on day 1 IP cisplatin 75mg/m2 on day 2 IP paclitaxel 40mg/m2 for 3h on day 8 (58.9%) 2) IP oxaliplatin and IV docetaxel (17.8%) 3) IP cisplatin 50-75mg/m2 (12.5%)	75% (43/56)	Median PFS: 10.5 months Median OS: 51 months	10.7% (6/56)
2	Milczek et al, 2012	74	N/A	Serous (100%)	Retrospecive, Double-arm (cisplatin , carboplatin group) Cohort study	1) Cisplatin group (66.2%, n=49) IP cisplatin 90mg/m2, IV cyclophosphamide on day 1 2) Carboplatin group (33.8%, n=25) IP carboplatin AUC 6, IV cyclophosphamide on day 1	100% (patients who completed all four IP cycles were chosen)	1) Median OS: 52 months 2) Cisplatin group Median OS: 59 months 3) Carboplatin group Median OS: 51 months	1) Bowel perforation during catheter insertion/removal: 10.8% (8/74) 2) Port obstruction: 8.1% (6/74)
3	Markman et al, 2009	432	N/A	Serous (67.9%)	Phase II clinical trials conducted by GOG	1) Cisplatin + 5-FU (GOG 102-B, 9.9%) 2) cisplatin + α-interferon (GOG 102-C, 9.7%) 3) cisplatin + etoposide (GOG 102-E, 19.4%) 4) α-interferon (GOG 102-F, 18.5%) 5) cisplatin + α-2b interferon (GOG 102-N, 12.7%) 6) paclitaxel (GOG 151, 17.5%)	N/A	Median OS: 28.8 months Median PFS: 14.4 months	N/A
4	Boisen et al, 2016	25	Platinum sensitive	Serous (84%)	Retrospective, Double-arm (longer TFI, shorter TFI after frontline therapy)	1) Cisplatin (n=20%) 2) Cisplatin + paclitaxel (n=80%)	1) shorter TFI (22 months): 13.3% (2/15) 2) longer TFI (31 months): 30.0% (3/10)	1) shorter TFI :16 months 2) longer TFI : 37 months	N/A

(Continued).

No.	Authors, Year	Enrolled patients	Platinum sensitivity	Pathology	Study Design	Drugs	Completion rate	Survival	Port related Complications
5	Recio et al, 1998	63	N/A	Serous (84%)	Retrospecitve, Single arm	Cisplatin 200mg/m2, cytarabine 1.2g/m2 (51%) + bleomycin 2U/m2 (49%)	N/A	Median OS: 29.1 months Median PFS: 9.6 months	port related peritonitis: 5% (3/63)
6	Lu et al, 2016	450	Platinum sensitive (328) resistant (122)	Serous (46.6%)	Retrospective, Double-arm (IP, IV group)	1) GOG 114 protocol (52.2%) IV carboplatin AUC 9 IV paclitaxel 135mg/m2 IP cisplatin 75mg/m2 2) GOG 172 protocol (45.5%) IV paclitaxel 135mg/m2 for 3h on day 1 IP cisplatin 100mg/m2 on day 2 IP paclitaxel 40mg/m2 for 3h on day 8	1) Platinum-sensitive Completion rate: 68% (73/108) 2) Platinum-resistant Completion rate: 62% (29/47)	1) Platinum-sensitive Median PFS: 9.8 months (IV control group : 6.9 months, p <0.001) 2) Platinum-resistant Median PFS: 4.9 months (IV control group : 2.4 months, p <0.001)	N/A

Abbreviations: IV, intravenous; IP, intraperitoneal; GOG, gynecologic oncology group; 5-FU,5-fluorouracil; OS, overall survival; PFS, progression free survival;.

Table S2. Experimental data with several cytotoxic drugs administered intraperitoneally on anastomotic healing.

No.	Authors, Year	Number of rats	Study Design	Drugs	Anastomotic bursting pressure
1	Kanellos et al, 2008	15	Experimental study	oxaliplatin 2.4 mg/kg control group: IP Nacl solution	N/A, Anastomotic leakage in 26.7% (0% in control group, p = 0.016)
2	Blouhos, 2010	30	Experimental study	oxaliplatin 2.4mg/kg control group: IP Nacl	183mmHg (300mmHg in control group <0.001)
3	Arikan et al, 2000	40	Experimental study	paclitaxel 3mg/kg control group: IP normal saline	126mmgHg (133mmHg in control group p<0.05)
4	Sasaya et al,1995	25	Experimental study	cisplatin 3mg/kg control group: IV cisplatin 3mg/kg	145mmHg (205mmHg in control group p<0.05)
5	Fumagalli, 1991	97	Experimental study	mitomycin-C 2mg/kg control group: IV mitomycin-C 1.5mg/kg	156mmHg (178mmgHg in control group p<0.01)

Abbreviation: IP, intraperitoneal; IV, intravenous.
